# A Case Resembling in Many Respects the Condition Known as Mucous Colitis

**Published:** 1898-06

**Authors:** E. G. Trevithick

**Affiliations:** Physician to the Cheltenham General Hospital


### A CASE RESEMBLING IN MANY RESPECTS THE CONDITION KNOWN AS MUCOUS COLITIS.

BY

E. G. Trevithick, M.A., M.D., B.C. Cantab., M.R.C.S. Eng.,

*Physician to the Cheltenham General Hospital.*

---

There is described in several text-books, most fully I think in those of American authorship,[1] a condition called mucous colitis, and the symptoms ascribed to this disorder fit in very well with those observed in the case I am about to narrate. But I gather

[1] See Osler's *Principles and Practice of Medicine*, 1892, pp. 396-7.

that those who have recorded cases have been definitely of the opinion that the tubular false membranes have been composed entirely of dense mucus. The microscopical appearance observed in the following case negatives the view that the case I met with was of this nature.

A.B., a spinster, aged 46, was admitted on November 15th, 1896. She was sent to the hospital by her private medical attendant as a case of appendicitis.

She gave the following history on admission:—Until a fortnight ago I was in my usual health. My appetite had been good and my bowels regular. On Saturday afternoon, a fortnight ago, I went out for a walk in the country. On my way home I felt very chilly. When I got home I felt so unwell that I went to bed. Two hours afterwards I was seized with severe griping pain in the right side. I felt sick, and vomited several times during the night. I had not been eating anything likely to disagree with me. I have been in bed ever since the attack, with severe pain in my stomach.

The following notes were then made:—Very depressed and emotional. Temperature, 100.4°. Pulse, 100. Very foul, moist tongue. Says her bowels have not been open for fourteen days. Cannot say whether her medicine has contained opium. Lies on back with thighs drawn up. There is considerable abdominal tenderness and rigidity. There is specially noticeable resistance in the right flank. No distinct abdominal tumour can be made out. *Per vaginam*, nothing abnormal can be detected. Urine very acid, s.g. 1018, showing distinct trace of albumen. Heart and lungs normal.

The diagnosis was uncertain. The vomiting, which had been a marked feature at the onset, had been getting less and less frequent. The condition in the flank was scarcely definite enough to lead us to suspect an appendicitis of fourteen days standing and of acute onset. There was something in the patient's demeanour which seemed to negative the existence of a perforation of the intestine. Her anxiety was very demonstrative, and had an air of fussiness about it. Nevertheless, there was evidence of some acute abdominal disorder, and the symptoms pointed to the intestine as the part primarily affected: as to the condition of the peritoneum, we felt uncertain. Notwithstanding the previous constipation, we thought it wise to give opiates. Small doses of morphia were ordered.

During the first five days after admission the temperature was irregular. On the evening of November 18th it reached 101°. That was the highest temperature recorded. On the sixth day the temperature was normal, nausea and vomiting had become altogether things of the past, and the abdominal rigidity and tenderness were much less. The patient was still markedly depressed and emotional. There had now been no action of the bowels for nearly three weeks. On the seventh day, emboldened by the general improvement, I suspended morphia, and ordered castor-oil and simple enemata, to which the bowels responded, but not copiously.

On December 3rd, fourteen days after admission, and twenty-eight days after the onset of her illness, the patient passed *per rectum*, together with a little bright blood, a greyish semi-translucent hollow cylindrical cast, seven inches long. Small doses of morphia were thereupon re-ordered.

She went on improving, and had no recurrence of hemorrhage and no return of pain. But eleven days after the passing of the first, she passed some more material of the same nature; and five days after that she passed another cylindrical piece, two inches long, inside which was fixed a scybalous mass of fæces.

She is at present in good health and good spirits, and is impressed with the idea that it is truly wonderful that she could ever have survived such a desperate illness as she seems to consider hers to have been.

When floated out in water the tube first passed, which some who saw it actually took at first sight to be a piece of intestine turned inside out, showed on its outer surface transverse ridges markedly suggestive of the folds of the mucous membrane of the small gut. The layer of which it was composed was about one-eighth of an inch thick. One end of the cylinder was well defined, and had a diameter of three-quarters of an inch. The other extremity was ragged, and showed a considerably larger lumen. In attempting to lift the cast from one vessel to another by slinging it over the handle of an ordinary bone penholder, it just broke in two of its own weight. The substance of which it was composed seemed, while floated in water, elastic and fairly firm, nor did the different parts of it show any tendency to stick together. But when a small fragment was removed and placed on a glass slide it lost its form, and behaved exactly like a little mass of rather dense mucus. When put into alcohol, a piece 3½ inches long rapidly lost weight, and became a shrivelled tube 1 in. long. It still, however, when thus shrunken showed the transverse ridges on its outer surface.

Microscopically the substance of which the cast is composed affords, in my opinion, evidence that originally it was a cellular formation. Coverslip preparations made by flattening out small fragments under slight pressure, when treated with hæmatoxylin, show well-defined, well-stained nuclei. In some places these appear thickly set in an almost structureless non-staining matrix. In others it is easy to make out the cell outlines, though these are for the most part imperfect. After repeated observations, I am forced to the belief that the substance in question consists of degenerated or semi-digested cells. Preparations stained with methyl blue show the whole to be swarming with micro-organisms, the predominating form being at any rate not unlike B. coli commune.

What could have been the cause and origin of this strange formation? To me there seemed at first sight three possibilities. (1) Had it been composed of parasitic amœboid cells? (2) Had it originally consisted of exuded leucocytes? Or (3) was it in reality a part of the intestine cast off, and altered by subsequent digestion and decomposition? This last mentioned view, I quickly discarded. But although I have examined many differently prepared microscopical specimens, I have still, day after day, found myself undecided between the first two alternatives. If the formation of this false membrane was in reality one of the results of a localised enteritis, then I must suppose that by the time it had reached us it was strangely altered. If it had once been fibrinous, it was no longer so.

---

